# 

**Published:** 1917-08-11

**Authors:** 


Rates of Subscription: Twelve months, 6/6; Six months, 4/-; three months, 2/-, post free to any address in the United Kingdom. Abroad, Twelve months, 8/8; Six months, 5/-; Three months, 2/6, post free. THE HOSPITAL "Index" to Volume LXI., October 1916 to March 1917, is now ready, price 2½d. per copy, post free. Address The Manager, The Hospital, "The Hospital" Building, 28/29 Southampton Street, Strand, London, W.C. 2.

**A NEW TREATMENT FOR GONORRHŒA.**

By CHARLES RUSS, M.B., M.R.C.S.,

*Physician-in-Charge, Electro-Therapeutic Department, Male Lock Hospital, London.*

Demy 8vo, 3s. net; post free, 3s. 4d.

"We have read this book with interest and profit, and cordially recommend it to the profession."—*The Medical Press.*

"This form of treatment merits further attention, as it is both scientific and ingenious."—*British Medical Journal.*

**A New Edition is in preparation.**

London: H. K. LEWIS & CO., Ltd., 136 Gower Street, W.C.

**UNIVERSITY OF LIVERPOOL.**

THE AUTUMN SESSION commences on October 9, 1917.

For information regarding all matters concerning the Curriculum, application should be made to the Dean.

Professor J. M. BEATTIE, M.D., *Dean.*

### Manager's Notices.

**Now Ready, "The Hospital" Index to Vol. LXI., Oct. 1916 to April 1917. Price 2½d. per copy post free.**

**All letters on business matters must be addressed to The Manager, "The Hospital," 28 and 29 Southampton Street, London, W.C. 2.**

Subscriptions, which may commence at any time, are payable in advance. Cheques and Post-Office Orders should be crossed London County and Westminster Bank, Covent Garden Branch, and made payable to the Manager of The Hospital.

Rates of Subscription (payable in advance).

| | |
|---|---|
| UNITED KINGDOM. | |
| Three Months (including Postage) | 2s. 0d |
| Six Months do. | 4s. 0d |
| Twelve Months do. | 6s. 6d. |
| FOREIGN AND COLONIAL. | |
| Three Months (including Postage) | 2s. 6d. |
| Six Months do | 5s. 0d. |
| Twelve Months do. | 8s. 8d. |

**Advertisements:**

To ensure insertion, all advertisements for The Hospital must reach the Manager not later than Wednesday at noon in each case.

*SUBSCRIPTION FORM.*

*Please send me* The Hospital *for* ............... *months, commencing with your issue of* ......................., *for which please find enclosed* ...........................

*Name* ...............................

*Address* ...............................

...............................

*Date* ...............................

*To* ............................... *Newsagent.*

